# Neighborhood Disadvantage and Allostatic Load in African American Women at Risk for Obesity-Related Diseases

**DOI:** 10.5888/pcd14.170143

**Published:** 2017-11-22

**Authors:** Marissa Tan, Abdullah Mamun, Heather Kitzman, Surendra Reddy Mandapati, Leilani Dodgen

**Affiliations:** 1 University of North Texas Health Science Center, Fort Worth, Texas; 2 Baylor Scott and White Health, Dallas, Texas

## Abstract

**Introduction:**

African American women have higher rates of obesity and related chronic disease than other demographic groups. The poorer health of African American women compared with other groups may be explained by allostatic load, or cumulative physiologic stress, due to chronic socioeconomic disadvantage. The objective of this study was to evaluate neighborhood and individual factors contributing to allostatic load in African American women at risk for obesity-related diseases.

**Methods:**

This study evaluated the relationship of allostatic load with neighborhood disadvantage, individual socioeconomic determinants, and synergism between neighborhood and socioeconomic disadvantage, along with health behaviors and other factors as mediators in African American women. Our sample consisted of 220 African American women at risk of obesity-related diseases enrolled in the Better Me Within program (mean [standard deviation] age, 50.1 [11.2] y; mean [standard deviation] body mass index, 36.7 [8.4] kg/m^2^). Allostatic load score for each participant was calculated by summing the number of biomarkers (of 9 biomarkers) that were determined to be in the high-risk quartile.

**Results:**

Poisson regression of neighborhood disadvantage and individual socioeconomic determinants found that neighborhood disadvantage, but not education level or household income, was significantly associated with allostatic load (β = 0.22, SE, 0.10, *P* = .04). Tests for mediators showed that household income and alcohol consumption partially mediated the relationship between allostatic load score and neighborhood disadvantage but were not significant.

**Conclusion:**

More research is necessary to determine the mechanisms by which neighborhoods can exacerbate and attenuate cumulative disadvantage among African American women. Policies and interventions that focus on neighborhood health may improve the outcomes of individual-level health interventions among women who reside in disadvantaged communities.

## Introduction

African American women have a higher prevalence of obesity (>50%) and associated chronic conditions than black men and non-Hispanic white men and women (1). The high prevalence of obesity may be associated with harmful coping behaviors used to manage the many roles that African American women are expected to fulfill in their daily lives (2). One clinical model that may explain these persistent health disparities is allostatic load, that is, the physiologic cost of cumulative stress (3). The concept of allostatic load is based in part on the weathering hypothesis, which attributes the poor health of African American women to chronic socioeconomic disadvantage (4). The inverse relationship between socioeconomic status (SES) and allostatic load in African American women may be due to poverty-related adversity and psychological responses to chronic stressors and disadvantage (5,6).

Although recent studies demonstrated the independent effect of neighborhood poverty on the relationship between low SES and the biomarkers of allostatic load in African Americans, limited research has focused exclusively on African American women (7,8). Neighborhood disadvantage in the context of allostatic load may be a proxy for exposure to multiple, chronic, stressful events; perceived safety; and access to health resources (9–11). One study of African American men and women found that health behaviors (diet, exercise, and smoking) partially mediated the relationship between neighborhood poverty and allostatic load (12). Another study of African Americans suggested that educational attainment, a component of individual SES, differentially affects the relationship between allostatic load and neighborhood disadvantage (13).

This study aimed to 1) expand the limited research on neighborhood disadvantage and low-income status, including possible synergism, on allostatic load and 2) investigate how education, income, health behaviors, and perceived stress influence allostatic load. We hypothesized that low-income status and neighborhood disadvantage independently and synergistically influence allostatic load and that education and health behaviors mediate the relationship.

## Methods

Trained staff members collected baseline data on 220 participants (from among 333 women who were screened for eligibility) before implementation of the Better Me Within cluster randomized controlled trial. The trial was conducted in 11 churches from February 2014 to May 2016 in Dallas, Texas, to test the efficacy of a church-based diabetes prevention program on weight reduction among overweight African American women. Participants resided in 148 census tracts in greater Dallas; most resided in Dallas County. Eligible participants self-identified as African American, were aged 18 years or older, had a body mass index (BMI, measured as weight in kilograms divided by height in meters squared [kg/m^2^]) of 25.0 or more, were not currently enrolled in another weight-loss program, attended an enrolled church, and did not have a health condition that restricted physical activity or altered their diet. Women with self-reported diabetes, a medical diagnosis of diabetes, or who had elevated fasting glucose (>126 mg/dL) and elevated hemoglobin A1c (>6.4%) were excluded. 

The institutional review board at The University of North Texas Health Science Center approved this study. All participants provided informed consent.

### Measures

**Neighborhood disadvantage.** Census tracts are small geographic areas that are used to predict small-area estimations of neighborhood deprivation (14). We examined 10 previously developed measures of neighborhood disadvantage: percentage of households living in poverty, percentage of households receiving public assistance, percentage of unoccupied housing units, percentage of renter-occupied housing, percentage of households living in the same house 5 years ago, percentage of occupied housing units with no vehicle, percentage of occupied housing units with more than 1 person per room (crowding), percentage of adults aged 25 or older without a high school diploma or equivalent, percentage of unemployed individuals 16 years or older in the civilian work force, and percentage of female-headed households (13). These data were collected from the 2015 American Community Survey, which determines poverty status by income, household size, and household members’ ages (15,16). For example, a 3-person household in 2015 with 1 member under age 18 and an annual household income below $19,043 is considered to be living in poverty (16). We used exploratory principal component analysis, a dimension-reduction technique, to create a composite socioeconomic score (13,17,18). The first principal component served as a composite score of neighborhood disadvantage that explained 49.9% of the variation. The median value of the first principal component was used to dichotomize the neighborhood of each participant as most disadvantaged or least disadvantaged (13). We used varimax orthogonal rotation to estimate the weights of the 10 neighborhood indicators used in the principal component analysis (Appendix A).

**Allostatic load score. **Of the various methods for measuring allostatic load, we selected the quartile method and 9 biomarkers: BMI, waist circumference, high-density lipoprotein (HDL) cholesterol, total cholesterol/HDL cholesterol ratio, triglycerides, glycosylated hemoglobin A1c (HbA1c), systolic blood pressure, diastolic blood pressure, and salivary cortisol (19). Height, measured with a stadiometer, and weight, measured with a medical-grade digital scale, were collected twice and averaged to calculate BMI. Waist circumference was measured (in duplicate and averaged) directly above the iliac crests with a tape measure by trained researchers. Data on HDL cholesterol, total cholesterol/HDL cholesterol ratio, triglycerides, and HbA1c were collected by using 2 fasting finger-stick samples and analyzed by using point-of-care tests on-site. Systolic and diastolic blood pressure was measured by using a standard automatic blood pressure cuff after the participant sat for 5 minutes; measurement was repeated after 3 minutes. Cortisol was measured by collecting a morning, fasting saliva sample and analyzed in a laboratory; cortisol measurement is a noninvasive way to measure physiologic stress (20). For each participant, we calculated an allostatic load score by summing the number of biomarkers for which the participant was categorized as high risk (19). A participant was categorized as high risk for a given biomarker if the biomarker value was in the highest quartile of our sample, except for HDL, for which the lowest quartile was considered high risk (Table 1). A participant’s biomarker was considered lower risk if the value was below the threshold of the highest quartile of the sample. Participants who reported current use of medications for hypertension, hypercholesterolemia, or prediabetes were categorized as high-risk for the corresponding biomarkers, regardless of their measurements (21).

**Table 1:** Mean, Median, Range, and Threshold of High-Risk Quartile for 9 Biomarkers of Allostatic Load at Baseline, Study of African American Women Participating in a Church-Based Diabetes Prevention Program on Weight Reduction (N = 220), Dallas, Texas, 2014–2016^a^

Variable	Mean (SD)	Median (Range)	High-Risk Quartile^b^
Systolic blood pressure, mm Hg	128.4 (19.3)	125.5 (97–216)	>138.5
Diastolic blood pressure, mm Hg	82.3 (10.8)	81.4 (56.6–120)	>88.5
HDL cholesterol, mg/dL	55.6 (13.9)	54 (27–100)	<46
Total cholesterol/HDL cholesterol ratio	3.3 (0.9)	3.52 (1.26–6.42)	>3.77
Hemoglobin A1c, %	6.0 (0.7)	5.9 (1.0–9.4)	>6.4
Body mass index, kg/m^2^	36.7 (8.4)	34.5 (25.0–84.6)	>40.6
Cortisol, ng/mL	2.7 (3.3)	2.1 (0.1–38.7)	>2.9
Waist circumference, in	41.3 (6.1)	40.4 (29.0–60.0)	>44.0
Triglycerides, mg/dL	113.4 (58.9)	95 (45–331)	>140

Abbreviation: HDL, high-density lipoprotein; SD, standard deviation.

a Missing values varied from 1% to 12% for the 9 biomarkers.

b A participant was categorized as high risk for a given biomarker if the biomarker value was in the highest quartile of our sample, except for HDL cholesterol, for which the lowest quartile was considered high risk. Quartiles were determined on the basis of data for each biomarker in our sample of 220 African American women.

**Individual socioeconomic variables.** Data on participants’ annual household income (categorized as <$25,000, $25,000–$49,999, $50,000–$74,999, and ≥$75,000) and highest level of educational attainment (categorized as ≤high school diploma or equivalent, some college or a technical degree, and college degree) were collected through self-reported surveys that used questions adapted from the Behavioral Risk Factor Surveillance System (BRFSS).

**Health behaviors.** Alcohol and tobacco use were measured through self-reported surveys by using questions adapted from the BRFSS and the National Institute on Alcohol Abuse and Alcoholism. Data on alcohol consumption was dichotomized as yes for participants who had at least 1 drink in the past 30 days and no for those who did not. Tobacco use was dichotomized as never for those who smoked fewer than 100 cigarettes in their lifetime and former or current for others. Physical activity data were collected from the Past Week Modifiable Physical Activity Questionnaire (22). Number of minutes of leisure-time physical activity was dichotomized according to meeting, or not meeting, guidelines of at least 150 minutes of weekly physical activity (23).

**Perceived stress.** Perceived stress was measured by using the 10-item Perceived Stress Scale in a self-report survey in which respondents reported feelings of stress and coping in the past month on a 5-point Likert scale from 0 (lowest) to 4 (highest) (24). The 10 items were summed to create a composite score for stress (score range, 0–40) in which greater values indicate greater levels of perceived stress.

### Statistical analysis

The distributions of education and income among individuals were assessed in the most disadvantaged neighborhoods and the least disadvantaged neighborhoods to examine mediating effects (Path B, Figure). We also assessed the distributions of health behaviors (alcohol consumption, smoking, and physical activity) and perceived stress between neighborhood types to examine mediating effects (Path B’, Figure). To test whether the relationship between neighborhood disadvantage and allostatic load was mediated by individual SES (education and income, Path C, Figure) and health behaviors (Path C’, Figure), we regressed allostatic load score on each factor separately. To determine the mediating effect of income on the relationship between allostatic load and neighborhood disadvantage, we regressed with an interaction term for synergistic effects.

**Figure:**
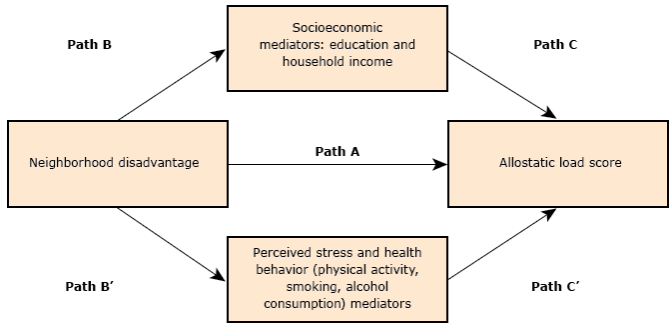
Hypothesized pathways mediating relationships between neighborhood disadvantage and allostatic load.

Approximately 12% (n = 26) of participants had missing values for some variables. Because a sensitivity analysis confirmed that data were missing at random, we used the Markov chain Monte Carlo method (25) to estimate a set of 20 imputed data for further analysis. We used SAS version 9.3 to analyze the data (SAS Institute Inc) with a 5% level of significance. We used PROC MI to estimate missing data and PROC GLIMMIX to create a 2-level mixed-effect model with a random intercept for each imputed data set. We used PROC MIANALYZE to estimate the effect of each variable on allostatic load score with a valid estimate of standard error (SE). Repeating the analysis with original data validated that imputation did not change the direction of results.

To assess the cluster effects of our data, we estimated the intraclass correlation coefficient as 0.12. The mean and variance of the outcome variable, allostatic load score, were close (2.34 and 2.87 respectively), satisfying the main assumption for Poisson regression for count data, which was confirmed by a goodness-of-fit test. A 2-level (hierarchical) Poisson regression model with a random intercept was used to estimate the effect of neighborhood disadvantage on allostatic load score after adjusting for demographic and health behavior variables. A 2-level negative binomial model to accommodate the model’s dispersion parameter did not alter the direction of results and showed similar effect sizes to the Poisson model and Akaike Information Criterion and Bayesian Information Criterion values. Ultimately, the Poisson model was performed for analyses (Appendix B).

## Results

The mean (standard deviation [SD]) age of participants was 50.1 (11.2) years, mean (SD) BMI was 36.7 (8.4), and mean (SD) waist circumference was 41.4 (6.1) inches (Table 2). The average age of participants living in each neighborhood type was similar.

**Table 2:** Descriptive Statistics for Individual and Neighborhood Variables^a^ at Baseline, By Neighborhood Type, Study of African American Women Participating in a Church-Based Diabetes Prevention Program on Weight Reduction (N = 220), Dallas, Texas, 2014–2016

Variable	All Neighborhoods (N = 220)	Most Disadvantaged Neighborhoods (n = 110)	Least Disadvantaged Neighborhoods (n = 110)	*P* Value
**Age, mean (SD), y**	50.1 (11.2)	50.1 (11.7)	50.1 (10.9)	.99^b^
**Body mass index, mean (SD), kg/m^2^**	36.7 (8.4)	37.6 (9.7)	35.7 (7.0)	.13^b^
**Waist circumference, mean (SD), in**	41.4 (6.1)	42.4 (6.2)	40.4 (5.9)	.06^b^
**Allostatic load score,^c^ mean (SD)**	2.3 (1.7)	2.7 (1.7)	2.0 (1.6)	.01^d^
**No. of high-risk biomarkers, no. (%) of participants**				
0	47 (22.1)	17 (16.0)	30 (28.0)	.02^d^
1–3	108 (50.7)	53 (50.0)	55 (51.4)	
>3	58 (27.2)	36 (34.0)	22 (20.6)	
**Composite neighborhood disadvantage score^e^, mean (SD)**	0 (1.0)	0.8 (0.7)	−0.8 (0.4)	<.001^b^
**Education, no. (%) of participants**				
≤High school diploma or equivalent	31 (15.5)	19 (19.4)	12 (11.8)	.39^f^
Some college/technical degree	74 (37.0)	40 (40.8)	34 (33.3)	
College degree	95 (47.5)	39 (39.8)	56 (54.9)	
**Annual household income, no. (%) of participants**				
<$25,000	40 (20.0)	27 (27.5)	13 (12.8)	.02^f^
$25,000–$49,999	64 (32.0)	34 (34.7)	30 (29.4)	
$50,000–$74,999	47 (23.5)	23 (23.5)	24 (23.5)	
≥$75,000	49 (24.5)	14 (14.3)	35 (34.3)	
**Physical activity, no. (%)**				
<150 min per week	141 (66.2)	70 (66.0)	71 (66.4)	.96^g^
≥150 min per week	72 (33.8)	36 (34.0)	36 (33.6)	
**Alcohol consumption in past 30 days, no. (%)**				
Yes	123 (57.7)	61 (57.6)	62 (58.0)	.95^g^
No	90 (42.3)	45 (42.4)	45 (42.0)	
**Smoking status, no. (%)**				
Never	163 (81.5)	78 (79.6)	85 (83.3)	.50^g^
Former/current	37 (18.5)	20 (20.4)	17 (16.7)	
**Perceived stress^h^, mean (SD)**	15.5 (6.8)	14.9 (6.7)	15.9 (7.2)	.30^b^

Abbreviation: SD, standard deviation.

a All data were measured at the individual level, except for composite neighborhood disadvantage score.

b *P* value obtained from hierarchical mixed-effect model for normal model with a random intercept.

c Calculated by summing the number of biomarkers for which the participant was categorized as high risk; score ranged from 0 to 9. Data were collected on 9 biomarkers: body mass index, waist circumference, high-density lipoprotein (HDL) cholesterol, total cholesterol/HDL cholesterol ratio, triglycerides, glycosylated hemoglobin A1c, systolic blood pressure, diastolic blood pressure, and salivary cortisol.

d *P* value obtained from hierarchical mixed-effect model for Poisson regression with a random intercept.

e Determined by examining 10 previously developed measures of disadvantage at the neighborhood level: percentage of households living in poverty, percentage of households receiving public assistance, percentage of unoccupied housing units, percentage of renter-occupied housing, percentage of households living in the same house 5 years ago, percentage of occupied housing units with no vehicle, percentage of occupied housing units with more than 1 person per room (crowding), percentage of adults aged 25 or older without a high school diploma or equivalent, percentage of unemployed individuals 16 years or older in the civilian work force, and percentage of female-headed households.

f *P* value obtained from hierarchical mixed effect model for multicategory logit model with a random intercept.

g *P* value obtained from hierarchical mixed effect model for logistic regression with a random intercept.

h Measured by using the 10-item Perceived Stress Scale in which respondents reported feelings of stress and coping in the past month on a 5-point Likert scale from 0 (lowest) to 4 (highest) (24). The 10 items were summed to create a composite score (score range, 0–40) for stress in which greater values indicate greater levels of perceived stress.

About one-fifth (22.1%) of participants had no high-risk biomarkers, and about half (47.5%) had a college degree. Because education was not significantly different between neighborhood types, we could not establish the mediating effects of individual SES (Path B, Figure). However, participants with higher income were clustered in the least disadvantaged neighborhoods (*F*_1, 194 _= 5.64, *P* = .02), which partially established a mediating effect (Path B, Figure). 

About one-third (33.8%) of participants reported 150 minutes or more of physical activity per week, which did not differ by neighborhood type (Table 2). Similar proportions in each neighborhood type consumed alcohol in the last 30 days and were current or former smokers. Perceived stress was approximately equal between neighborhood types. Therefore, health behavior and perceived stress variables did not establish a mediating effect (Path B’, Figure).

The most disadvantaged neighborhoods had significantly higher percentages of households living in poverty, households receiving public assistance, unoccupied housing units, renter-occupied housing, households without a vehicle, crowding, adults aged 25 or older without a high school diploma or equivalent, and unemployed individuals age 16 or older, compared with the least disadvantaged neighborhoods (*P* < .001 for each) (Table 3). The percentage of households who lived in their homes for 5 or more years and the percentage of female-headed households were similar between the most and least disadvantaged neighborhoods.

**Table 3:** Descriptive Statistics for Components of Neighborhood Disadvantage, by Neighborhood Type, at Baseline, Study of African American Women Participating in a Church-Based Diabetes Prevention Program on Weight Reduction (N = 220), Dallas, Texas, 2014–2016

Component	Total Sample Mean (SD)	Most Disadvantaged Neighborhoods Mean (SD)	Least Disadvantaged Neighborhoods Mean (SD)	*P* Value^a^
Percentage of households living in poverty	19.7 (12.9)	28.5 (11.7)	10.9 (6.2)	<.001
Percentage of household receiving public assistance	32.8 (18.6)	44.65 (15.5)	21.0 (13.0)	<.001
Percentage of unoccupied housing units	8.8 (6.2)	11.6 (6.5)	5.9 (4.5)	<.001
Percentage of renter-occupied housing	43.9 (24.4)	51.3 (21.9)	36.5 (24.6)	<.001
Percentage of households living in the same house in past 5 years	60.6 (16.1)	60.3 (13.4)	60.9 (18.5)	.76
Percentage of occupied housing units with no vehicle	8.8 (9.0)	13.6 (10.0)	3.9 (3.8)	<.001
Percentage of occupied housing units with >1 person per room (crowding)	4.9 (4.2)	7.4 (4.2)	2.4 (2.2)	<.001
Percentage of adults 25 years or older without a high school diploma or equivalent	20.1 (12.9)	30.1 (10.4)	10.0 (5.0)	<.001
Percentage of unemployed individuals aged 16 years or older in the civilian labor force	8.9 (5.2)	11.8 (5.8)	6.1 (2.3)	<.001
Percentage of female-headed households	18.3 (4.8)	18.5 (4.5)	18.2 (5.0)	.56

Abbreviation: SD, standard deviation.

a *P* value obtained from hierarchical mixed-effect model for normal model with a random intercept.

After we adjusted for participant age, we found a significant positive association between the most disadvantaged neighborhood and allostatic load score (Model 1: β = 0.24; SE, 0.10; *P* = .02, Table 4), which established the effect hypothesized as Path A (Figure). In this model, women living in the most disadvantaged neighborhoods had a 1.3-unit higher allostatic load score on average than women living in the least disadvantaged neighborhoods. In Model 2, after adjustment for age, individual socioeconomic factors, and a synergistic effect of neighborhood disadvantage and income, the association between neighborhood disadvantage and allostatic load score was no longer significant (Table 4, Model 2). This result may have occurred because of the multicollinearity introduced by the interaction term. However, income showed a trend for lower allostatic load score with higher levels of income. After adjustment for health behaviors and perceived stress in Model 3, neighborhood disadvantage (β = 0.25; SE, 0.10; *P* = .02) and alcohol consumption (β = −0.23; SE, 0.10; *P* = .02) were significant. In the full model (Model 4), which adjusted for socioeconomic characteristics and health behaviors, alcohol consumption (β = −0.20; SE, 0.10; *P* = .07) was no longer significant, and neighborhood disadvantage remained significant, although slightly weakened (β = 0.22; SE, 0.10; *P* = .04).

**Table 4:** Adjusted Association of Allostatic Load Score with Neighborhood and Individual Variables at Baseline, Poisson Regression, Study of African American Women Participating in a Church-Based Diabetes Prevention Program on Weight Reduction (N = 220), Dallas, Texas, 2014–2016

Variable	β (SE) [*P* Value]			
	Model 1	Model 2	Model 3	Model 4
**Intercept**	−0.10 (0.23) [.67]	−0.04 (0.33) [.89]	−0.15 (0.31) [.62]	−0.13 (0.34) [.70]
**Neighborhood disadvantage**				
Most disadvantaged	0.24 (0.10) [.02]	0.21 (0.22) [.33]	0.25 (0.10) [.02]	0.22 (0.10) [.04]
Least disadvantaged	1 [Reference]	1 [Reference]	1 [Reference]	1 [Reference]
**Age, y**	0.02 (0.004) [.001]	0.02 (0.004) [<.001]	0.01 (0.004) [.004]	0.01 (0.004) [.003]

**Socioeconomic Mediators**				
**Annual household income, $**				
<25,000	—	1 [Reference]	—	1 [Reference]
25,000–49,999	—	−0.15 (0.23) [.52]	—	−0.13 (0.13) [.31]
50,000–74,999	—	−0.26 (0.24) [.29]	—	−0.20 (0.15) [.18]
≥75,000	—	−0.29 (0.23) [.22]	—	−0.22 (0.16) [.19]
**Effect of interaction between neighborhood disadvantage and income**				
Least disadvantaged and <$25,000	—	1 [Reference]	—	—
Least disadvantaged and $25,000–$49,999	—	−0.03 (0.27) [.90]	—	—
Least disadvantaged and $50,000–$74,999	—	−0.02 (0.30) [.83]	—	—
Least disadvantaged and ≥ $75,000	—	−0.03 (0.30) [.92]	—	—
**Education**				
≤High school	—	1 [Reference]	—	1 [Reference]
Some college/technical degree	—	0.26 (0.14) [.08]	—	0.26 (0.15) [.08]
College degree	—	0.07 (0.15) [.66]	—	0.06 (0.15) [.70]

**Health Behaviors and Perceived Stress Mediators**				
**Alcohol consumption in past 30 days**				
Yes	—	—	−0.23 (0.10) [.02]	−0.20 (0.10) [.07]
No	—	—	1 [Reference]	1 [Reference]
**Physical activity**				
<150 min per week	—	—	0.12 (0.10) [.23]	0.11 (0.10) [.30]
≥150 min per week	—	—	1 [Reference]	1 [Reference]
**Smoking**				
Current/former	—	—	−0.03 (0.12) [.83]	−0.10 (0.13) [.46]
Never	—	—	1 [Reference]	1 [Reference]
**Perceived stress**	—	—	0.01 (0.01) [.15]	0.01 (0.01) [.22]
**σ^2^**	0.04 (0.03)	0.04 (0.03)	0.04 (0.03)	0.04 (0.03)

Abbreviation: SE, standard error.

## Discussion

In this sample of African American women at risk for obesity-related diseases, after adjustment for socioeconomic and health behavior variables, our results partially support one of our hypotheses: that living in a disadvantaged neighborhood is associated with higher allostatic load. This finding is consistent with previous research showing that neighborhood disadvantage is associated with higher allostatic load in African Americans (13,17). Previous studies of African American women also showed that women residing in areas of greater neighborhood poverty had higher cumulative biological risk than women living in less impoverished neighborhoods (8). Conversely, individuals living in high-income neighborhoods had lower cumulative biological risk than those who live in low-income neighborhoods (12). Our study adds to this research by evaluating health behaviors and individual socioeconomic factors as mediators in the relationship between neighborhood disadvantage and allostatic load exclusively in African American women who are at risk for obesity-related diseases.

Although neither a significant mediator nor synergism between low-income status and neighborhood disadvantage was discovered in the final model, we found nonsignificant trends of lower allostatic load for both alcohol consumption in the past 30 days and higher household income. These trends may indicate that household income and alcohol consumption partially mediate the relationship between neighborhood disadvantage and allostatic load. Harmful health behaviors that are used for coping with high levels of stress (eg, low levels of physical activity, smoking) were not significantly associated with allostatic load in our study.

That household income did not significantly predict allostatic load is consistent with the findings of Barber et al (13). Our findings contrast with those of several studies of neighborhood poverty and cumulative biological risk; these studies found household income to be an independent and significant predictor among women (7,8,12). However, the association of higher household income with lower allostatic load in our study, although not significant, suggests that women may weather both individual and neighborhood socioeconomic disadvantage.

The lack of a significant relationship between educational attainment and allostatic load reflects a complex relationship. Some studies found that low educational attainment and low income were associated with high allostatic load (5,17). In contrast, another study found that the relationship of neighborhood disadvantage and cumulative biological risk had a weaker association among African Americans who did not finish high school than among those who had (13). Other studies found differences in allostatic factor loading (eg, metabolic vs inflammatory) by level of educational attainment (26). Contextual factors, like segregation, may also influence the relationship between education and health; this idea is supported by our finding that neighborhood disadvantage is associated with higher allostatic load (27).

Our study found an inverse relationship between alcohol consumption in the past 30 days and allostatic load. This finding is consistent with research showing that light and moderate levels of drinking are associated with lower levels of heart disease and diabetes (28). Another study found that alcohol consumption was inversely related to allostatic load (7). Future research should investigate how levels of alcohol consumption influence allostatic load; our study provides only general information on alcohol consumption.

Our study has several strengths. To our knowledge, ours is the first study to focus on the neighborhood and individual determinants of allostatic load in a sample of African American women at risk for obesity-related diseases. Our sample population was relatively homogenous demographically, and it excluded the effect of diabetic disease processes on allostatic load biomarkers. Our allostatic load score comprised 9 biomarkers, which reflect anthropomorphic, neuroendocrine, cardiovascular, and metabolic domains of allostatic load. By testing mediators through stepwise models, we minimized the potential for artificial correlations.

Our study has several limitations. We could not establish a causal relationship between neighborhood disadvantage and allostatic load because of the study’s cross-sectional design. We did not collect data on the length of participants’ residence at their current address or previous residence in a disadvantaged neighborhood; previous residence in a disadvantaged neighborhood is associated with higher rates of cardiometabolic disease than never having left a neighborhood or never having lived in a poor neighborhood (29). The study had a relatively small sample size (N = 220), compared with the sample sizes other studies, which may have limited the power to identify mediating variables such as education. Our measure of physical activity was self-reported and did not distinguish between levels of intensity (30). We were also unable to compare our data with data on other races and ethnicities because we focused exclusively on African American women. However, research focusing on overweight African American women, who are at higher risk for obesity-related chronic diseases compared with other groups, is sparse. Lastly, our measure of alcohol consumption did not evaluate dose, but rather any alcohol intake in the past 30 days, limiting our ability to draw conclusions on this variable.

Despite these limitations, our study has implications for future research on neighborhood disadvantage among African American women. The consistent relationship of neighborhood disadvantage with allostatic load warrants investigation of the social and physical environment of disadvantaged neighborhoods to elucidate the mechanisms by which women accumulate physiologic stress. Several important components of the physical environment for obesity-related health disparities have been identified and may influence allostatic load: accessibility of food stores, exercise facilities, and hospitals; sidewalks; and safety (11,14,). One study in Dallas showed that a change in the previous year’s crime rate was associated with higher levels of C-reactive protein, an inflammatory marker, in women (10).

Our cross-sectional findings provide evidence to inform future longitudinal studies on the effects of community-based interventions on allostatic load in African American women. By evaluating these interventions in the context of neighborhood disadvantage, we can determine the individual and neighborhood variables that can mitigate the effects of chronic disadvantage on the health of African American women. Overall, our study adds to the body of knowledge on neighborhood effects on allostatic load in African American women and demonstrates the crucial need for health equity policies that prevent and reduce the health risks associated with living in disadvantaged neighborhoods.

## Appendix A Varimax Orthogonal Rotated Weight (Standardized) for the First Principal Component of the Neighborhood Indicators at Baseline, Study of African American Women Participating in a Church-Based Diabetes Prevention Program on Weight Reduction (N = 220), Dallas, Texas^a^

**Table:**

Neighborhood Indicator	Weight
Percentage of households living in poverty	0.22
Percentage of household receiving public assistance	0.17
Percentage of housing units unoccupied	0.07
Percentage of renter-occupied housing	−0.03
Percentage of households living in the same house in past 5 years	0.14
Percentage of occupied housing units with no vehicle	0.12
Percentage of occupied housing units with >1 person per room (crowding)	0.23
Percentage of adults 25 years or older with <high school diploma or equivalent	0.31
Percentage of unemployed individuals 16 years or older in the civilian labor force	0.19
Percentage of female-headed households	−0.13

^a ^Six participants were missing census tract data, so for this analysis, n = 214.

## Appendix B Two-Level Poisson Regression Model Used to Perform Regression Analyses

Level 1 (between-subjects effect):

ln (θ*_ij_*) = β_0_
*_j_* + β_1_
*_j_ D_ij _* + ∑β*_cj_*
*X_cij_*
(1)

*Y_ij_* ~ Poisson(θ*_ij_*) (2)

In equation 2, *Y_ij_* represents allostatic load score that follows Poisson distribution with mean θ*_ij_* for subject *i* in church *j* (*j* = 1, 2, …, 11). In equation 1, β_0_ *_j_* is the adjusted mean allostatic load score in natural logarithm base for church *j* after controlling for all variables. The adjusted effect of neighborhood (*D_ij_*) and other variables (*X_cij_*) on allostatic load score are estimated using β_1_ *_j_* and β*_cj_* in natural logarithm base as fixed effect at level-1 for church *j*.

Level 2 (cluster effect):

β_0_
*_j_* = γ_00_ + *u*_0_
*_j_*,

β_1_
*_j_* = γ_10_ + *u*_0_
*_j_*
(3)

β*_cj_* = γ*_c_*_0_, *c* = 1, 2, …, *c*

In equation 3, γ_00_ is the overall adjusted mean allostatic load score at natural logarithm base after controlling for all variables. γ_10 _and γ*_c_*_0_ are the pooled within-church regression coefficients at natural logarithm base for the level-1 covariates, and *u*_0_ *_j _* is an error term representing effects associated with church *j*, which is assumed to be normally distributed with mean 0 and variance 1. Here, *c* represents the number of covariates in the model.
